# Surface Integrity of Widely Used Resin-Modified Glass Ionomers: New Findings Amid Limited Scientific Literature

**DOI:** 10.4317/jced.64010

**Published:** 2026-05-29

**Authors:** Kelyn Ticona-Canavera, José Giancarlo Tozo-Burgos

**Affiliations:** 1 Research Group on Dental Biomaterials and Natural Products, Faculty of Health Sciences, Universidad Privada de Tacna, 23000 Tacna, Peru

## Abstract

**Background:**

Resin-modified glass ionomer cements are considered bioactive materials due to their sustained fluoride release, which has supported their continued use in clinical practice. Although some of these materials have been available on the market for many years and continue to be widely used in restorative dentistry, the scientific evidence regarding their mechanical properties remains limited and, in many cases, based on older or insufficiently updated studies. This situation highlights the need to generate new and updated scientific evidence to better understand the behavior of these materials under conditions that simulate long-term clinical use. The aim of this study was to evaluate and compare the surface microhardness of three resin-modified glass ionomer cements after an artificial aging process simulating two years of clinical use.

**Materials and Methods:**

Thirty-six discs (10 mm × 2 mm) were fabricated and distributed into three experimental groups (n = 12), corresponding to three commercial resin-modified glass ionomer cements: Gold Label 2 LC, Riva LC, and Vitremer. All specimens were prepared strictly according to the manufacturers' instructions. Surface microhardness was evaluated using the Vickers method with a calibrated microhardness tester, performing three indentations per specimen. Measurements were carried out at three experimental time points: baseline, after 10,000 cycles, and after 20,000 thermocycles. Data were analyzed using descriptive statistics, analysis of variance, and a linear mixed-effects model, considering a significance level of = 0.05.

**Results:**

All evaluated materials showed a progressive decrease in surface microhardness after artificial aging. Statistically significant differences were observed among the resin-modified glass ionomer cements (p &lt; 0.05). Gold Label 2 LC exhibited the highest surface microhardness values and the greatest stability throughout the aging process, followed by Riva LC, whereas Vitremer showed the lowest microhardness values after aging.

**Conclusions:**

After artificial aging, all resin-modified glass ionomer cements showed a progressive decrease in surface microhardness. Gold Label 2 LC exhibited the highest stability, followed by Riva LC, whereas Vitremer showed the least favorable performance.

## Introduction

Resin-modified glass ionomer cements (RMGICs) represent an important advancement in restorative dentistry compared with conventional glass ionomer cements, as they combine the characteristic acid-base reaction of these materials with free-radical polymerization of monomers such as 2-hydroxyethyl methacrylate (HEMA), typical of conventional resin systems (1 , 2). This dual-curing mechanism provides clinically relevant advantages, including early mechanical strength, reduced sensitivity to moisture during setting, and improved esthetic properties, bringing their performance closer to that of composite resins (3). One of the most remarkable properties of RMGICs is their ability to release fluoride in a sustained manner, which promotes remineralization and acts as a preventive factor against secondary caries. Additionally, they exhibit chemical and micromechanical adhesion to enamel and dentin, adequate wear resistance, and greater clinical retention when compared with conventional glass ionomer cements (4 , 5). Nevertheless, the incorporation of resin also introduces certain limitations. Polymerization shrinkage and a higher coefficient of linear thermal expansion may negatively affect marginal integrity in long-term restorations. Furthermore, the release of HEMA has been associated with potential cytotoxic effects and increased water sorption, which may compromise the mechanical stability of the material over time. These factors highlight the importance of proper manipulation protocols and optimal light-curing procedures to ensure satisfactory clinical performance (6). In clinical practice, RMGICs are frequently used in direct restorations of Class III and V cavities, in pediatric dentistry, as pit and fissure sealants, and as cavity bases. In these indications, they combine their bioactive potential with the mechanical properties of conventional resin-based materials, offering a versatile alternative for minimally invasive restorative approaches (7). Among resin-modified glass ionomer cements, one of the most widely used products in clinical practice is Vitremer (3M). Since its introduction to the market, several in vitro studies have demonstrated its favorable marginal adaptation, in some cases comparable to that observed with materials such as mineral trioxide aggregate (MTA) (8). Its clinical applicability has also been reported in specific procedures, such as furcation sealing when used in combination with a collagen sponge, showing satisfactory handling and sealing ability (9). In addition, Vitremer stands out for its high fluoride-release capacity, reported to be greater than that of other compomers, with an effect that tends to increase over time as the material matures, reinforcing its cariostatic potential (10). A clinical study evaluating restorations in non-carious cervical lesions compared Vitremer with a conventional composite resin and reported a 100% retention rate for Vitremer after two years of follow-up, whereas composite resin restorations showed a retention rate of 78.8% (11). Conversely, in vitro investigations have shown that although Vitremer is capable of releasing fluoride in a sustained manner for up to 84 days in acidic environments, this bioactivity may be accompanied by a marked reduction in mechanical properties, including losses greater than 50% in flexural strength and elastic modulus (12). Complementary evidence has demonstrated a significant decrease in surface microhardness after short-term immersion in solutions with neutral and acidic pH, confirming that while bioactivity is preserved, physico-mechanical properties may be adversely affected by environmental conditions (13). The restorative materials market currently offers a wide range of RMGICs. Some have been available for many years but are supported by limited scientific evidence, whereas others have been introduced more recently and are promoted as superior despite being supported by a relatively small number of studies. One representative example is Fuji II LC (GC), a light-cured resin-modified glass ionomer cement indicated for Class III and V restorations, primary teeth, and cavity bases. The most representative clinical evaluation of this material is the trial conducted by Perdigão et al. (14), in which three restorative options for non-carious cervical lesions were compared. After six months and one year of follow-up, both RMGICs showed 100% retention, whereas the composite resin presented a retention rate of 92.6%. Regarding surface behavior, Fuji II LC exhibited greater deterioration than the composite resin but less than Ketac Nano, which showed higher staining and poorer marginal adaptation among the evaluated materials. Similarly, an in vitro study assessed the Vickers microhardness of esthetic restorative materials, including Fuji II LC (GC) and Vitremer (3M ESPE), at different depths from the light-cured surface toward the interior of the material. The results demonstrated that both RMGICs maintained stable microhardness values at all analyzed depths, whereas compomers and composite resins showed a significant reduction in hardness as depth increased, suggesting differences in polymerization efficiency and structural stability (15). Another commercially available alternative is Riva LC (SDI), which, according to the manufacturer, offers enhanced mechanical strength and sustained fluoride release compared with conventional formulations. However, scientific evidence supporting its performance remains limited. Rêgo et al. (16) compared Riva LC with other RMGICs and reported similar flexural and fracture resistance to Fuji II LC, but superior fluoride release compared with the other evaluated materials. In a complementary study, Ramos et al. (17) evaluated several physico-mechanical properties of restorative materials, with particular emphasis on Riva LC (SDI). This material was assessed in terms of microhardness, flexural strength, sorption, solubility, and dentin bond strength and compared with conventional composite resins and the alkasite-type material Cention N. The results indicated that Riva LC showed acceptable and intermediate performance within its category, with values close to those of reference materials with established mechanical properties, although lower than those observed for composite resins. To evaluate the long-term behavior of restorative materials, artificial aging procedures are widely used, as they allow controlled simulation of oral environmental conditions and prediction of clinical performance (18). Thermal cycling, in particular, is known to induce surface alterations associated with degradation processes occurring in the oral cavity, such as hydrolytic degradation and mechanical fatigue (19 , 20). Owing to this approximation to clinical reality, artificial aging by thermal cycling is recognized in the literature as a valid and reliable method for assessing the durability of restorative materials (21 , 22). Therefore, generating updated evidence on the performance of RMGICs remains necessary, considering that long-term clinical success depends on the stability of their physico-mechanical properties. Surface microhardness is of particular relevance, as it is closely related to wear resistance and restoration longevity. Given the limited and heterogeneous evidence currently available, the present study was justified, with the aim of evaluating and comparing the surface microhardness of three resin-modified glass ionomer cements after artificial aging equivalent to two years of clinical use.

## Materials and Methods

- Study design, ethics, and sample size. This in vitro experimental study, with a prospective and longitudinal design, was approved by the Ethics Committee of the Universidad Privada de Tacna (FACSA-CEI/170-11-2025). The sample size was calculated using G*Power software (version 3.0.10), applying a repeated-measures ANOVA with within- and between-subjects interaction. Three experimental groups corresponding to three resin-modified glass ionomer cements (RMGICs) and three evaluation time points (initial, after 10,000 and 20,000 thermocycling cycles) were considered. A medium effect size (f = 0.25), a significance level of 0.05, a statistical power of 80%, and a correlation among repeated measurements of 0.5 were assumed, resulting in a total sample of 36 specimens distributed into three groups of 12 specimens each. - Sample preparation Discs measuring 10 mm in diameter and 2 mm in thickness were fabricated for each resin-modified glass ionomer cement brand (12 specimens per group), according to the materials described in Table 1.

Table 1

**Figure 1:**
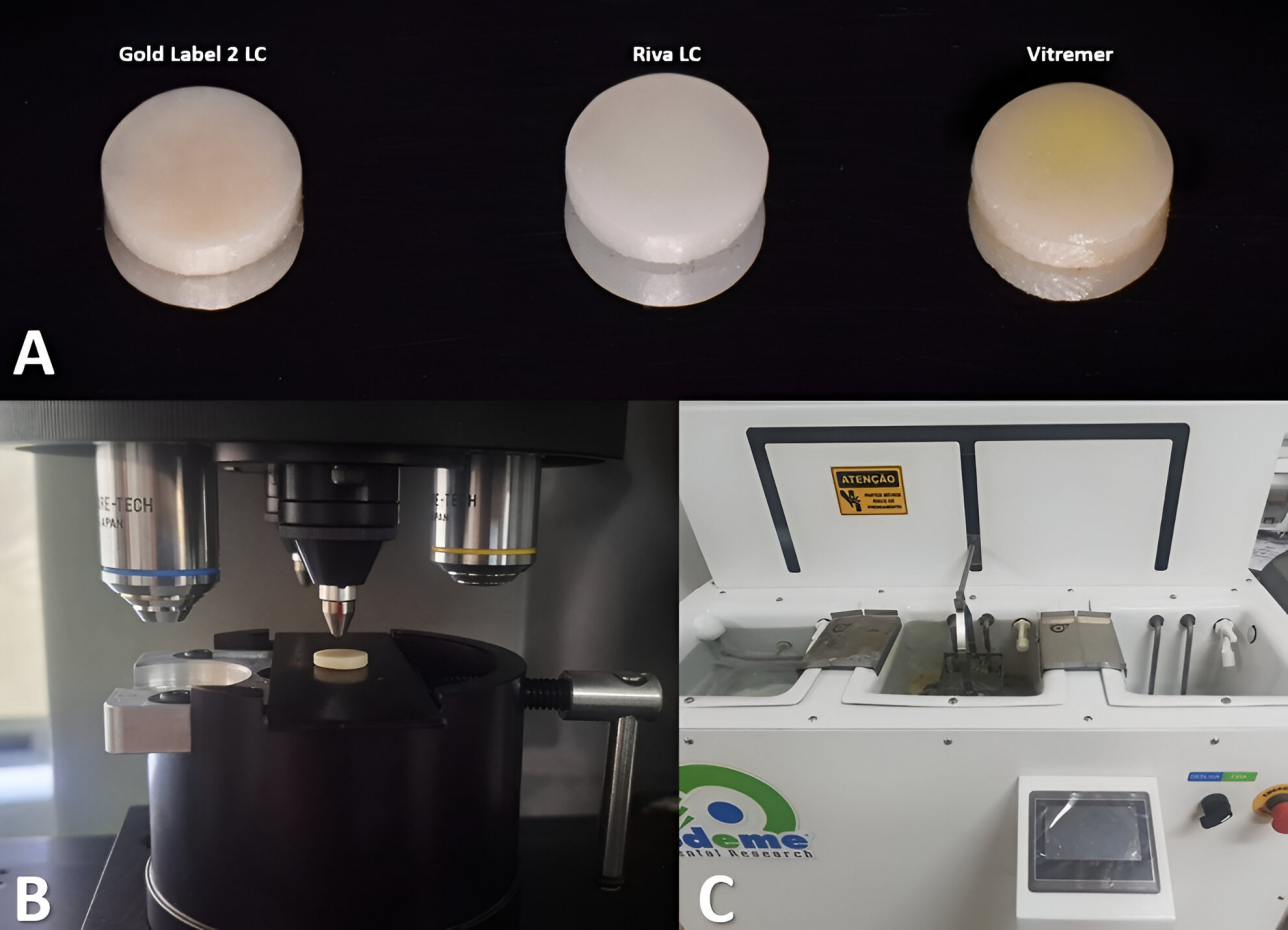
Experimental procedure, (A) RMGIC discs from each brand, (B) Surface microhardness test, (C) Thermocycling-induced artificial aging.

The materials were selected due to their widespread clinical use, as well as their differences in composition, setting mechanisms, and physicochemical behavior, and because, although some of them have been used in clinical practice for many years, there is still limited or insufficiently updated scientific evidence regarding their mechanical properties. Specimen preparation was performed under controlled laboratory conditions, using nitrile gloves and previously disinfected surfaces to minimize the risk of contamination. The fabrication method and specimen dimensions were based on a previously published study (23), in which stainless steel metal molds with standardized circular cavities were used. Prior to material placement, the cavities were lubricated with a thin layer of glycerin applied using a microbrush to facilitate specimen removal. The preparation of the resin-modified glass ionomer cements was carried out carefully and strictly in accordance with the manufacturers' instructions, respecting the exact proportions recommended for each of the three experimental groups corresponding to the brands listed in Table 1. All specimens were light-cured using a VALO LED curing unit (Ultradent Products Inc., South Jordan, Utah, USA), with a constant irradiance of 1,000 mW/cm², maintained perpendicular to the surface at an approximate distance of 1 mm, for 20 or 40 seconds (depending on the material used) on each side of the disc, ensuring homogeneous curing conditions across all experimental groups. After polymerization, the specimens were carefully removed from the molds to avoid damage to edges and surfaces. Subsequently, their dimensions were verified using a high-precision digital caliper (±0.01 mm), and specimens that did not meet the standardized dimensions or exhibited visible surface irregularities were discarded. Finishing and polishing of the discs were performed immediately after fabrication using the Sof-Lex disc system under water cooling (3M ESPE, St. Paul, MN, USA). The three available grit sizes (medium, fine, and superfine) were applied sequentially at reduced speed to obtain homogeneous, smooth, and reproducible surfaces, in accordance with protocols previously reported in the literature (24). Finally, in the group corresponding to the Vitremer system, the proprietary surface coating (Vitremer Finishing Gloss) was applied to both sides of the discs following the manufacturer's recommendations, in order to simulate natural clinical conditions, considering that the other evaluated systems do not include this procedure in their finishing protocol (Fig. 1A).

1

- Surface microhardness test Surface microhardness was evaluated using the Vickers method with a microhardness tester (AMH55; LECO, St. Joseph, MI, USA), following protocols previously described for this type of material. A Vickers diamond pyramidal indenter was used. Three indentations were performed on each specimen at randomly selected points around the central region of the surface. Indentations were placed at least 1 mm apart and at a minimum distance of 1 mm from the specimen margins to avoid interaction effects. Measurements were carried out by applying a load of 100 g for 10 seconds (25). The obtained values were recorded and subsequently averaged to determine the final microhardness value for each specimen. Prior to each measurement, the specimens were gently dried with absorbent paper to remove excess surface moisture. The device was calibrated according to the manufacturer's instructions before testing. This procedure was repeated at three experimental time points: before artificial aging, after 10,000 cycles, and after 20,000 thermocycling cycles (Fig. 1B). - Artificial aging process Artificial aging was performed using an automated thermocycling process in an OMC-350 TS unit (Odeme Dental Research, Brazil), equipped with two temperature-controlled water baths set at 5°C ± 2°C and 55°C ± 2°C, following protocols previously described in the literature. Each cycle consisted of alternate immersion of the specimens for 40 seconds in each bath, with a transfer time of 15 seconds between baths (25). The procedure was carried out until completion of 10,000 and 20,000 thermocycling cycles (Fig. 1C). It was considered that 10,000 and 20,000 thermocycling cycles correspond approximately to one and two years of clinical service, respectively, as reported in previous studies, based on the assumption that dental materials are exposed to 20-50 thermal changes per day, although this equivalence should be interpreted as an approximation (26). -Statistical Analysis Data were analyzed using STATA software, version 19.0 (StataCorp LP, College Station, TX, USA). Descriptive analysis of surface microhardness values was performed and expressed as mean and standard deviation. Data normality was assessed using the Shapiro-Wilk test, and homogeneity of variances was verified using the Breusch-Pagan/Cook-Weisberg test. Differences in surface microhardness among the three resin-modified glass ionomer cements after artificial aging were analyzed using one-way analysis of variance (ANOVA). When statistically significant differences were identified, post hoc multiple comparisons were performed using the Bonferroni adjustment. Additionally, changes in surface microhardness throughout the artificial aging process (initial, 10,000, and 20,000 thermocycling cycles) were evaluated using a linear mixed-effects model, considering material type and evaluation time as fixed effects and specimen as a random effect. The level of statistical significance was set at = 0.05.

## Results

Table 2 illustrates a progressive reduction in surface microhardness for all three materials as the number of aging cycles increased.

Table 2

Gold Label 2LC consistently exhibited the highest microhardness values at all evaluation time points, followed by Riva LC, whereas Vitremer showed the lowest values throughout the study. A decrease in microhardness from the initial measurement to 20,000 thermocycling cycles was observed for all materials, demonstrating the effect of artificial aging on the surface resistance of the evaluated glass ionomer cements. Table 3 presents the results of the analysis of variance comparing Vickers microhardness values among the different materials.

Table 3

Statistically significant differences were observed between groups (p = 0.008), with a moderate effect size (² = 0.23), indicating that material type accounted for 23% of the observed variability. Bonferroni post hoc analysis identified statistically significant differences only between Vitremer and Gold Label 2LC (p = 0.007). Table 4 presents the results of the linear mixed-effects model, which showed a significant decrease in Vickers microhardness (HV) associated with aging.

Table 4

**Figure 2:**
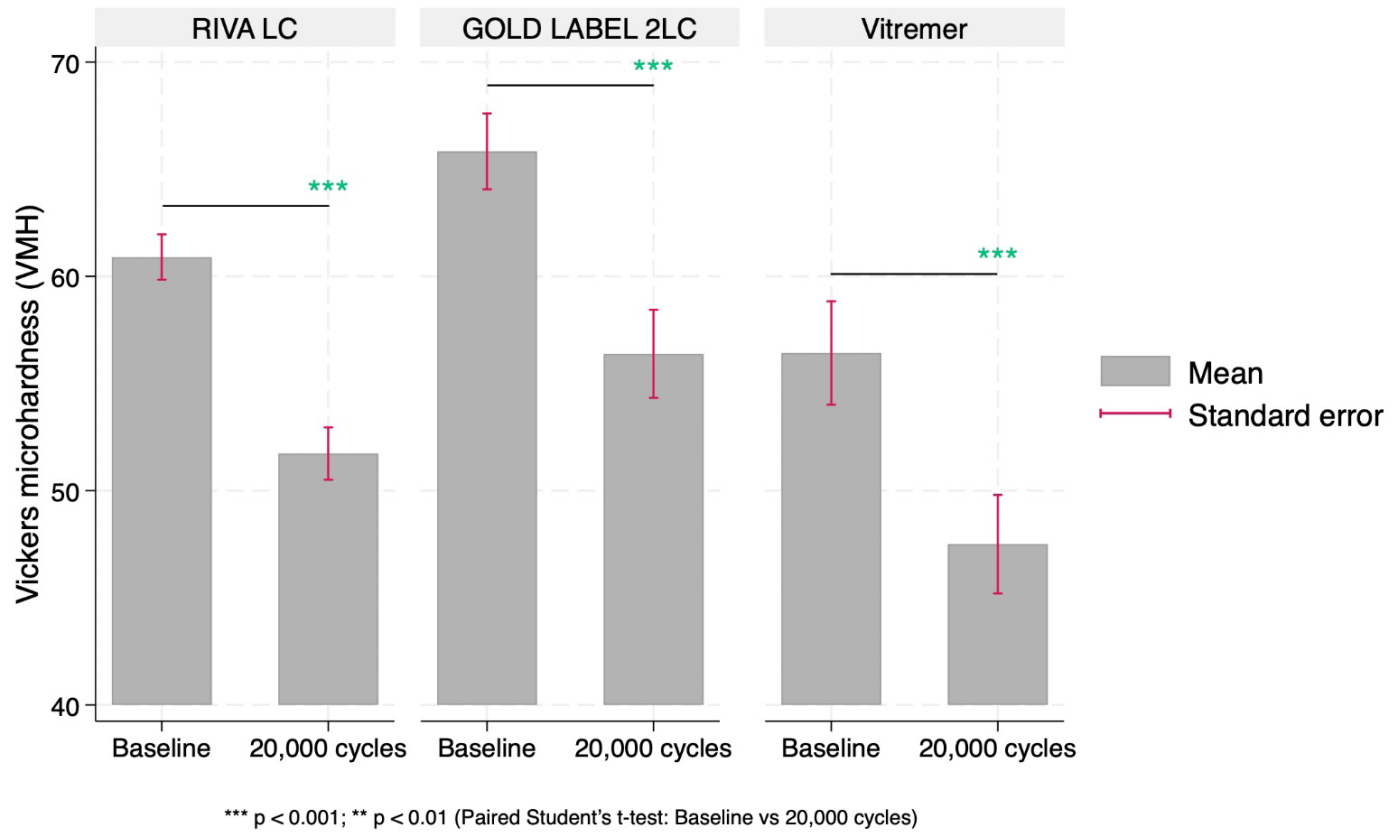
Changes in Vickers microhardness of resin-modified glass ionomer cements after artificial aging.

Significant reductions were observed at both 10,000 cycles ( = 3.67; p = 0.009) and 20,000 cycles ( = 9.17; p &lt; 0.001) when compared with initial values. Additionally, a further significant decrease was identified between 10,000 and 20,000 cycles ( = 5.50; p &lt; 0.001). With respect to material type, Gold Label 2LC exhibited significantly higher microhardness values than Riva LC ( = 4.93; p = 0.041). Vitremer showed a tendency toward lower microhardness values compared with Riva LC, although this difference did not reach statistical significance (p = 0.064), and significantly lower values when compared with Gold Label 2LC ( = 9.40; p &lt; 0.001). No significant interaction was observed between aging time and RMGIC type (p &gt; 0.05), indicating that the pattern of microhardness reduction over time was similar across the evaluated materials. Figure 2 illustrates the comparison between initial Vickers microhardness and values obtained after 20,000 aging cycles for each evaluated material.

2

A statistically significant reduction in microhardness was observed in Riva LC (p &lt; 0.001) as well as in Gold Label 2LC (p&lt; 0.001). Likewise, Vitremer showed a significant decrease in microhardness after aging (p = 0.002). Overall, these results indicate that artificial aging led to a significant deterioration in microhardness across all evaluated materials.

## Discussion

As previously noted, resin modified glass ionomer cements (RMGICs) are widely used in clinical practice. However, the available scientific evidence has primarily focused on the evaluation of their bioactive properties, such as fluoride release and remineralization potential, with variable results among different materials (4 , 5). In contrast, information regarding the surface stability and mechanical behavior of these materials remains limited. This gap underscores the relevance of the present study, which evaluated the surface degradation of three commercial RMGICs (Gold Label 2 LC, Riva LC, and Vitremer) using an artificial aging protocol based on thermocycling equivalent to two years of clinical use, and its impact on surface microhardness values. The results demonstrated a progressive decrease in surface microhardness in all three evaluated materials. A significant reduction was observed after the first 10,000 thermocycling cycles, which became more pronounced after 20,000 cycles, revealing a consistent trend across all materials. These findings confirm the detrimental effect of thermal aging on the surface mechanical stability of RMGICs. The present results are consistent with those reported by Birant and Gumustas (25), who evaluated the effect of thermal aging on the surface and mechanical properties of various bioactive restorative materials, including RMGICs, high viscosity glass ionomers, glass carbomer cement, and resin based bioactive materials such as bioactive composites and alkasites, using a comparable thermocycling protocol. The authors demonstrated that thermocycling induces significant surface alterations, manifested as reduced microhardness and topographical changes associated with thermal stress and water absorption. Although that study represents one of the few direct references evaluating similar materials under comparable experimental conditions, aging was limited to 10,000 cycles, approximately equivalent to one year of clinical use, and materials with different structural matrices were included. In that context, the alkasite material Cention N exhibited the best mechanical performance after aging. In the present study, Gold Label 2 LC consistently exhibited the highest surface microhardness values at all experimental time points, as well as the greatest resistance to artificial aging by thermocycling. This behavior may be attributed to specific features of its formulation and setting mechanism. Gold Label 2 LC is a powder liquid RMGIC in which the acid base reaction predominates in matrix formation, complemented by a light curing phase that reinforces internal cohesion. This balance may favor the development of a more integrated microstructure that is less dependent on additional surface coatings, unlike other systems that require protective layers for initial stability. Furthermore, the possibility of immediate finishing and polishing suggests sufficient early consolidation, potentially reducing susceptibility to water uptake and thermally induced degradation, which is reflected in the preservation of surface microhardness values after aging. Although Gold Label 2 LC has been used clinically for several years, scientific evidence specifically addressing its mechanical behavior remains scarce. To the best of current knowledge, only one in vitro study has included this material in a comparative analysis alongside other restorative materials such as Biodentine and flowable composites, reporting fracture resistance values close to 250 N, comparable to the evaluated materials (27). In this context, it is reasonable to interpret the present findings in light of evidence available for other RMGICs from the same manufacturer, such as Fuji II LC, which has demonstrated stable and predictable mechanical behavior in various experimental settings. Previous studies have shown that Fuji II LC exhibits superior mechanical properties compared with other RMGICs, including Riva LC, as evidenced by flexural strength, fracture toughness, and diametral tensile strength tests, maintaining acceptable microhardness values even after aging procedures (15 , 25). Riva LC is another RMGIC with limited scientific evidence, possibly due to its relatively more recent introduction to the market. This scarcity of studies has constrained a comprehensive understanding of its long term mechanical behavior. Among the available reports, Schwendicke et al. (28) demonstrated that Riva LC promotes consistent mineral gain in artificial dentin caries lesions under various demineralization conditions, confirming its bioactive potential. However, these mineral gains were not always associated with proportional increases in microhardness, suggesting that its remineralizing effect may be primarily related to ionic release and dentin substrate stabilization. Similarly, Panpisut and Toneluck (29) evaluated several physico mechanical and functional properties of restorative materials, including Riva LC, and reported a high degree of monomer conversion and the highest cumulative fluoride release. Nevertheless, this behavior was accompanied by increased water sorption and volumetric expansion, as well as lower flexural strength values, indicating a trade off between bioactivity and mechanical performance favoring the bioactive component. Consistent with these observations, the present study showed a progressive reduction in surface microhardness for Riva LC, suggesting that the water affinity that supports its bioactivity may partially compromise surface stability over time. Despite this, Riva LC exhibited an intermediate performance compared with the other evaluated materials. In contrast, Vitremer demonstrated the least favorable performance, exhibiting lower Vickers microhardness values from the initial measurement and after both 10,000 and 20,000 thermocycling cycles. This behavior may be primarily explained by its high water affinity and the hydrophilic nature of its resin matrix. Cefaly et al. (30) reported significantly higher water sorption for Vitremer compared with other RMGICs, attributing this behavior to the presence of HEMA and a lower degree of polymer cross linking, factors that promote matrix plasticization and surface mechanical degradation. These findings were corroborated by Beriat and Nalbant (31), who observed the highest HEMA release and water absorption for Vitremer among evaluated RMGICs, with a positive correlation between both phenomena. Likewise, Lima et al. (32) reported high water sorption and solubility values for Vitremer, even when prepared strictly according to manufacturer instructions. It is noteworthy that in those studies, sample preparation was generally described according to manufacturer recommendations, without specifying the application of the proprietary Vitremer surface coating. In contrast, the present study included the application of the Finishing Gloss coating; nevertheless, a progressive decrease in microhardness was still observed, suggesting that the observed behavior is primarily related to intrinsic material properties rather than the finishing protocol. Despite the application of the protective coating, the decrease in microhardness suggests that intrinsic factors of the material, such as its high-water affinity and hydrophilic resin matrix, play a predominant role over surface protection. From a microstructural perspective, Guedes et al. (33) reported more irregular surfaces and higher fluoride concentrations in Vitremer using SEM and EDX analyses, which, while enhancing bioactivity, may increase susceptibility to surface degradation under conditions of moisture and repeated thermal stress. Taken together, the available evidence indicates that the balance between bioactivity and mechanical performance in Vitremer is strongly shifted toward bioactivity. Although high ionic release and water affinity are beneficial from an anticariogenic standpoint, they predispose the material to progressive surface instability following aging. Among the limitations of this study, its in vitro design should be acknowledged. Nevertheless, artificial aging by thermocycling is a widely accepted methodology for simulating thermal changes experienced by restorative materials in the oral environment. Surface microhardness was evaluated using calibrated microhardness testers, a validated and widely employed technique for assessing surface mechanical behavior. Additionally, specimen preparation required strict control of mixing and handling procedures, as variations in powder liquid ratios can influence material properties; however, all samples were prepared in strict accordance with manufacturer instructions, supporting the reliability of the findings. However, this study did not include mechanical loading, pH variations, or biofilm simulation, factors that may influence the clinical performance of these materials. Future studies could complement these results through microstructural analyses or additional mechanical tests to further elucidate material behavior under conditions that more comprehensively simulate the oral environment.

## Conclusions

The present study demonstrated that all three evaluated resin modified glass ionomer cements exhibited a progressive reduction in surface microhardness after artificial aging. Among the materials tested, Gold Label 2 LC showed the highest surface stability throughout the aging process, followed by Riva LC, which displayed intermediate performance. In contrast, Vitremer presented the least favorable behavior, showing greater susceptibility to surface degradation over the entire experimental period.

## Figures and Tables

**Table 1:** Resin modified glass ionomer cements included in the study.

Commercial name	Composition	Manipulation	Batch
Gold Label 2 LC GC Corporation, Tokyo, Japan	Powder: Fluoroaluminosilicate glass, inorganic fillers and pigments. Liquid: Polyacrylic acid modified with HEMA, hydrophilic monomers, water, and photoinitiator system.	Powder liquid system. 1 level scoop of powder: 2 drops of liquid. Mixing time: 20 seconds until a homogeneous consistency is obtained. Light curing: 20 seconds per surface.	2410081
Riva LC SDI Limited, Bayswater, Australia	Powder: Reactive fluoroaluminosilicate glass and inorganic fillers. Liquid: Polyacrylic acid modified with resin, hydrophilic methacrylate monomers, water, and photoinitiator system.	Powder liquid system. 1 level scoop of powder: 2 drops of liquid. Mixing time: 20 to 30 seconds. Light curing: 20 seconds per surface.	1250033
Vitremer 3M ESPE, St. Paul, MN, USA	Powder: Fluoroaluminosilicate glass with lanthanum, potassium persulfate, ascorbic acid, pigments, and inorganic fillers. Liquid: Polyacrylic acid modified with HEMA, dimethacrylate monomers, water, and photoinitiator system.	Powder liquid system. Manufacturer recommended proportion (scoop and liquid volume supplied in the kit). Mixing time: 45 seconds. Light curing: 40 seconds per surface. Subsequent application of Vitremer Finishing Gloss and additional light curing according to the manufacturer.	11512090

1

**Table 2:** Vickers microhardness (mean ± SD) of the resin modified glass ionomer cements evaluated at different aging time points.

Material	Initial	10,000 cycles	20,000 cycles
Riva Lc	60.90 ± 3.81	57.23 ± 4.00	51.73 ± 4.40
Gold Label 2lc	65.83 ± 6.37	61.50 ± 6.35	56.38 ± 7.40
Vitremer	56.42 ± 8.69	50.72 ± 6.36	47.50 ± 8.27

2

**Table 3:** Analysis of variance (ANOVA) for the comparison of Vickers microhardness among the evaluated materials after the aging process.

Source	SS	df	MS	F	p value	η²
Between groups	513.47	2	256.73	5.39	0.008	0.23
Within groups	1713.68	36	47.60			

3

**Table 4:** Effect of aging and material type on Vickers microhardness.

Variable	Comparison	β estimate	95% CI	p value
Time	10,000 cycles vs Initial	-3.67	-6.43; -0.91	0.009
	20,000 cycles vs Initial	-9.17	-11.93; -6.41	<0.001
	10,000 cycles vs 20,000 cycles	-5.5	-8.26; -2.73	<0.001
Material	Gold Label 2LC Vs Riva LC	4.93	0.19; 9.67	0.041
	Vitremer vs Riva LC	-4.48	-9.22; 0.26	0.064
	Vitremer vs Gold Label 2LC	-9.40	-14.14; -4.66	<0.001
Interaction	Time × Material	−	−	ns

Note: Results were obtained using a linear mixed-effects model with specimen as a random effect. β represents the mean change in Vickers microhardness (HV) relative to the reference category. ns: not significant

## References

[1] de Gee AJ, Leloup G, Werner A, Vreven J, Davidson CL (1998). Structural integrity of resin- modified glass ionomers as affected by the delay or omission of light activation. J Dent Res.

[2] Dursun E, Nguyen JF, Tang ML, Attal JP, Sadoun M (2016). HEMA release and degree of conversion from a resin-modified glass ionomer cement after various delays of light activation. Dent Mater.

[3] Berzins DW, Abey S, Costache MC, Wilkie CA, Roberts HW (2010). Resin-modified glass-ionomer setting reaction competition. J Dent Res.

[4] Hicks J, Garcia-Godoy F, Donly K, Flaitz C (2003). Fluoride-releasing restorative materials and secondary caries. J Calif Dent Assoc.

[5] Visel D, Jäcker T, Jost-Brinkmann PG, Präger TM (2014). Demineralization adjacent to orthodontic brackets after application of conventional and self-etching primer systems. J Orofac Orthop.

[6] Lim HN, Kim SH, Yu B, Lee YK (2009). Influence of HEMA content on the mechanical and bonding properties of experimental HEMA-added glass ionomer cements. J Appl Oral Sci.

[7] Ge KX, Lam WYH, Chu CH, Yu OY (2024). Updates on the clinical application of glass ionomer cement in restorative and preventive dentistry. J Dent Sci.

[8] Costa AT, Konrath F, Dedavid B, Weber JBB, de Oliveira MG (2009). Marginal adaptation of root-end filling materials: an in vitro study with teeth and replicas. J Contemp Dent Pract.

[9] Tsatsas DV, Meliou HA, Kerezoudis NP (2005). Sealing effectiveness of materials used in furcation perforation in vitro. Int Dent J.

[10] Marks LA, Verbeeck RM, De Maeyer EA, Martens LC (2000). Effect of maturation on the fluoride release of resin-modified glass ionomer and polyacid-modified composite resin cements. Biomaterials.

[11] Santiago SL, Passos VF, Vieira AHM, Navarro MFL, Lauris JRP, Franco EB (2010). Two-year clinical evaluation of resinous restorative systems in non-carious cervical lesions. Braz Dent J.

[12] Moreau JL, Xu HHK (2010). Fluoride releasing restorative materials: effects of pH on mechanical properties and ion release. Dent Mater.

[13] Silva KG, Pedrini D, Delbem ACB, Cannon M (2007). Microhardness and fluoride release of restorative materials in different storage media. Braz Dent J.

[14] Perdigão J, Dutra-Corrêa M, Saraceni SHC, Ciaramicoli MT, Kiyan VH (2012). Randomized clinical trial of two resin-modified glass ionomer materials: 1-year results. Oper Dent.

[15] Palma-Dibb RG, Palma AE, Matson E, Chinelatti MA, Ramos RP (2003). Microhardness of esthetic restorative materials at different depths. Mater Res.

[16] Rêgo HMC, Butler S, Santos MJC (2022). Evaluation of the mechanical properties of three resin-modified glass-ionomer materials. Biomed Res Int.

[17] Ramos NBP, Felizardo KR, Berger SB, Guiraldo RD, Lopes MB (2024). Comparative study of physical-chemical properties of bioactive glass ionomer cement. Braz Dent J.

[18] Pieniak D, Niewczas A, Walczak A, Łępicka M, Grądzka-Dahlke M, Maciejewski R (2020). The effect of thermal stresses on the functional properties of various dental composites. Tribol Int.

[19] Krüger J, Maletz R, Ottl P, Warkentin M (2018). In vitro aging behavior of dental composites considering the influence of filler content, storage media and incubation time. PLoS One.

[20] Teixeira GS, Pereira GKR, Susin AH (2021). Aging methods: an evaluation of their influence on bond strength. Eur J Dent.

[21] Szczesio-Wlodarczyk A, Kopacz K, Ranoszek-Soliwoda K, Sokolowski J, Bociong K (2025). Towards the standardization of artificial aging protocols for dental composites: evaluation of proposed methods. J Funct Biomater.

[22] Szczesio-Wlodarczyk A, Sokolowski J, Kleczewska J, Bociong K (2020). Ageing of dental composites based on methacrylate resins: a critical review of the causes and method of assessment. Polymers (Basel).

[23] Ismail HS, Ali AI, Abo El-Ella MA, Mahmoud SH (2020). Effect of different polishing techniques on surface roughness and bacterial adhesion of three glass ionomer- based restorative materials: in vitro study. J Clin Exp Dent.

[24] Miličević A, Goršeta K, van Duinen RN, Glavina D (2018). Surface roughness of glass ionomer cements after application of different polishing techniques. Acta Stomatol Croat.

[25] Birant S, Gümüştaş B (2024). The effect of thermal aging on microhardness and SEM/EDS for characterisation bioactive filling materials. BMC Oral Health.

[26] Gale MS, Darvell BW (1999). Thermal cycling procedures for laboratory testing of dental restorations. J Dent.

[27] D’Souza JM, de Ataide I de N, Lambor R (2024). Effect of newer intraorifice barriers on the fracture resistance of endodontically treated teeth: An in vitro study. Cureus.

[28] Schwendicke F, Al-Abdi A, Pascual Moscardó A, Ferrando Cascales A, Sauro S (2019). Remineralization effects of conventional and experimental ion-releasing materials in chemically or bacterially-induced dentin caries lesions. Dent Mater.

[29] Panpisut P, Toneluck A (2020). Monomer conversion, dimensional stability, biaxial flexural strength, and fluoride release of resin-based restorative material containing alkaline fillers. Dent Mater J.

[30] Cefaly DFG, Wang L, de Mello LLCP, dos Santos JL, dos Santos JR, Lauris JRP (2006). Water sorption of resin-modified glass-ionomer cements photoactivated with LED. Braz Oral Res.

[31] Beriat NC, Nalbant D (2009). Water absorption and HEMA release of resin-modified glass-ionomers. Eur J Dent.

[32] Lima RBW, Farias JFG, Andrade AKM, Silva FDSCM, Duarte RM (2018). Water sorption and solubility of glass ionomer cements indicated for atraumatic restorative treatment considering the time and the pH of the storage solution. Rev Gaucha Odontol.

[33] Guedes OA, Bandeca MC, Nakatani MK, de Araújo Estrela CR, de Alencar AHG (2015). Chemical and structural characterization of glass ionomer cements indicated for atraumatic restorative treatment. J Contemp Dent Pract.

